# Sustainable Soil Washing: Shredded Card Filtration of Potentially Toxic Elements after Leaching from Soil Using Organic Acid Solutions

**DOI:** 10.1371/journal.pone.0149882

**Published:** 2016-02-22

**Authors:** Christopher Ash, Ondřej Drábek, Václav Tejnecký, Jan Jehlička, Ninon Michon, Luboš Borůvka

**Affiliations:** 1 Department of Soil Science and Soil Protection, Faculty of Agrobiology, Food and Natural Resources, Czech University of Life Sciences, Prague, Czech Republic; 2 Department of Environmental Geosciences, Faculty of Environmental Sciences, Czech University of Life Sciences, Prague, Czech Republic; Institute for Sustainable Plant Protection, C.N.R., ITALY

## Abstract

Shredded card (SC) was assessed for use as a sorbent of potentially toxic elements (PTE) carried from contaminated soil in various leachates (oxalic acid, formic acid, CaCl_2_, water). We further assessed SC for retention of PTE, using acidified water (pH 3.4). Vertical columns and a peristaltic pump were used to leach PTE from soils (O and A/B horizons) before passing through SC. Sorption onto SC was studied by comparing leachates, and by monitoring total PTE contents on SC before and after leaching. SC buffers against acidic soil conditions that promote metals solubility; considerable increases in solution pH (+4.49) were observed. Greatest differences in solution PTE content after leaching with/without SC occurred for Pb. In oxalic acid, As, Cd, Pb showed a high level of sorption (25, 15, and 58x more of the respective PTE in leachates without SC). In formic acid, Pb sorption was highly efficient (219x more Pb in leachate without SC). In water, only Pb showed high sorption (191x more Pb in leachate without SC). In desorption experiments, release of PTE from SC varied according to the source of PTE (organic/mineral soil), and type of solvent used. Arsenic was the PTE most readily leached in desorption experiments. Low As sorption from water was followed by fast release (70% As released from SC). A high rate of Cd sorption from organic acid solutions was followed by strong retention (~12% Cd desorption). SC also retained Pb after sorption from water, with subsequent losses of ≤8.5% of total bound Pb. The proposed use of this material is for the filtration of PTE from extract solution following soil washing. Low-molecular-mass organic acids offer a less destructive, biodegradable alternative to strong inorganic acids for soil washing.

## Introduction

Contamination of the environment with potentially toxic elements (PTE) is a persistent threat in the industrialized world. Remedial responses to soils polluted with PTE vary according to many factors, such as the contamination source, and the pathways or environmental media through which PTE must pass before reaching a target [1].

Column leaching experiments are a commonly used tool for the simulation of a given solution percolating through a porous medium [2]. To a certain extent, they allow the user to determine the level of binding and mobility of PTE through applied leaching of the solution of interest. Metals dissolved in the extract solution represent an environmental hazard, and metals left in the soil following leaching are likely to be present in chemically stable mineral forms and bound to non-labile fractions [3].

In soils, conventional remedial techniques are typically based on containment of contaminants, or removal of contaminated soil for treatment on or off site. Certain agents are chosen to enhance the solubilization of inorganic contaminants in soil; following decontamination, the selected agent must be recovered, and possibly treated. In general, bases and complexing agents are rarely applied because of the difficulties of treating the wastewater [4]. Strong mineral acids such as HCl, and HNO_3_ can be used to leach PTE from soil, however, these strong acids can compromise soil structure. Alternatively, weaker organic acids may be equally as effective in the removal of PTE, whilst at the same time being easily decomposed by microorganisms following washing, and maintaining desirable soil properties. Many studies have focused on the use of amendments to immobilize and reduce availability of PTE through their incorporation into the soil. Amendments can be mixed into the contaminated soil or into a layer that forms a barrier between the pollution source and the environmental receptor. Houben et al. [5] studied several cost effective amendments for their potential immobilization of Cd, Pb and Zn. Calcium carbonate, iron grit, fly ash, manure, bentonite and bone meal all proved effective at reducing Cd and Zn leaching, mainly due to the increase in pH that was caused by amendment additions. However, only CaCO_3_ addition resulted in a strong reduction of plant uptake for the considered metals. Recycling of materials with adsorbent properties for the immobilization of toxic metals offers obvious benefits. Consequently, studies have focused on using a range of materials for this purpose, including simple amendments such as sawdust [6],[7],[8] and brewers’ draff [9], and more comparatively hi-tech materials such as recycled activated alumina and recycled collagen fiber [10].

A relatively high anionic charge density of mechanical pulp fibers is consistent with the presence of high levels of resin acids, fatty acids, hemicellulose, and certain degradation products of lignin [11]. Furthermore, the addition of calcium carbonate as a binding agent during production gives paper and card products a highly alkaline property (pH > 8.0). Research by Prica et al. [12] focused on the potential for cardboard mill pulp as an immobilization amendment to Cd enriched sediments, making use of standardized leaching tests using weak acetic acid and humic acid solutions. In earlier works, Battaglia et al. [13] and Calace et al. [14] also experimented with paper mill sludge for the treatment of metal polluted soils.

The aim of this study was to analyze different leaching solutions (oxalic acid, formic acid, CaCl_2_, deionized water) for content of As, Cd, and Pb after passing through columns of contaminated soil with/without a shredded cardboard (SC) filter. We then further assess SC for its subsequent effectiveness at retaining PTE after leaching with acidified deionized water. We propose that SC could be applied as a filter to remove PTE from the extract solution following soil washing.

## Methodology

### Sample collection and pre-treatment

Soil samples were collected from a forested area near to Přibram in the Czech Republic (49.7°N, 14.0°E) that has been heavily affected by atmospheric deposition of Cd, Pb, and As [15]. A map of the area can be seen in S1 Fig. No specific permissions were required for the access and sampling of the location used, nor did the field study involve endangered or protected species. Two separate samples were collected from a single soil pit. The upper layer (3–11.5 cm), a mixture of F and H organic horizons was labeled as ‘soil O’. This represents a strongly contaminated soil rich in organic matter. In the underlying layers, a shallow organo-mineral A horizon (11.5–13.5 cm) and mineral B horizon (13.5–20 cm) were taken as a mixed sample and labeled as ‘soil AB’. This sample represents a well-developed mineral soil with some organic constituents, which is also contaminated. On return to the laboratory, samples were air dried (~25°C) to constant weight.

### Soil analyses

Soil pH was measured by preparing a 1:5 soil:liquid (w:v) ratio using water (pH_H2O_) or 2 M KCl solution (pH_KCl_) then measuring after agitation using a Denver Instrument UB-5 pH meter. Content of combustible soil organic matter (Corg) was measured by the loss on ignition method. Quality of humic substances was calculated from spectrophotometric analysis, taken as the ratio of a pyrophosphate soil extract absorbance at wavelengths 400 and 600 nm (_A400/A600_) [16]. Cation exchange capacity (CEC) was determined according to the Bower method as described by Hesse [17], using Varian Spectra AA280 FS (Mulgrave, Australia). Elemental analysis of N, C, S was made using a Thermo Scientific Flash 2000 NCS Analyzer. All analyses were made in triplicates.

Total contents of PTE in 1g of soil and SC samples was determined by digestion in a mixture of concentrated acids (HF, HNO_3_), with heating on a hot plate for 16 hrs at 190°C. After evaporation, 1 ml of 65% HNO_3_ and 49 ml of deionized water (DI_H2O_) were added to the residue before filtering (Whatman 390). Extracts were analyzed by inductively coupled plasma-optical emission spectrometer (ICP-OES, iCAP 7000 Duo ICP; Thermo Scientific) under standard conditions. Blanks, and standard reference material “Montana II” and “San Joaquin” by NIST, which represent polluted and baseline soils respectively, were used to control analytical performance.

### Leaching experiment

#### Adsorption

This study focuses on the possible use of shredded cardboard (SC) as a sorbent material to capture PTE following soil washing. Cardboard used was salvaged generic brown packaging card that was free of plastic tape, ink or print. Cardboard was ground in a mill to obtain a light porous shred. Some physicochemical properties of the SC are given in Table 1.

**Table 1:** Physicochemical properties and total PTE content of soil and SC samples.

	pH		Organic matter		CEC	N	C	Total content mg/kg		
	pH_H2O_	pH_KCl_	Corg (humus)	Q _A400/A600_	cmol/kg	%		As	Cd	Pb
Soil O	4.17±0.01	3.66±0.04	77.0%(nd)	8.10	95±13.3	1.70±0.02	28.1±0.05	266±16.6	14.4±0.05	24962±1401
Soil AB	4.27±0.01	3.61±<0.01	22.4%(5.80%)	7.26	46.5±11.7	0.31±0.01	4.60±0.10	327±20.0	5.86±0.24	5046±255
SC	8.27±0.03	8.03±0.01	-	-	16.3±0.40	0.18±<0.01	38.6±0.05	0.42±0.36	0.14±0.02	7.06±5.40

Corg = combustible organic matter, Q _4/6_ = ratio of a pyrophosphate soil extract absorbance at wavelengths 400 and 600 nm (lower Q _4/6_ value corresponds to a greater degree of humification). CEC = cation exchange capacity. nd = no data. Analyses were performed in triplicates

For use as the mobilizing solvent, this study focuses on the following low-molecular-mass organic acids (LMMOA): 1 mM formic acid (pKa 3.75) and 1 mM oxalic acid (pKa_1_ 1.25, pKa_2_ 3.81). LMMOA were produced by Lach-Ner, s.r.o. Czech Republic. An unbuffered salt solution of 0.1 M CaCl_2_, a solvent commonly used to represent availability and mobility of metals in polluted soils evaluation [18], and DI_H2O_ (not acidified), which represents a control solution, were also included in the leaching study.

Soil samples were ground and sieved (< 2 mm) to obtain homogeneous material. Vertical glass columns (18 x 3 cm) were used to contain soil. Either side of the sample (layer equal to 5 g of soil) was a filter disk. A layer of small glass marbles was used to separate soil sample from SC, which was positioned in the column ~2 cm above the soil sample. SC was also added on a weight basis (5 g). An illustration of the experimental layout can be viewed (S2 Fig). Experimental solutions were pumped vertically upward through the column; solution mobility was provided by a peristaltic pump (Kouřil PCD 83.4S) set to flow at 100 mL/hr, giving an hourly solution:soil (LS) ratio of 20:1 (v:w). Leaching experiments ran continuously for 24 hrs. After 24 hrs, collected solutions were filtered through nylon 0.45 mm syringe filters, and then target elements were measured using ICP-OES. Content of anions were also determined using an ion chromatograph ICS 1600 equipped with IonPac AS11-HC. Tejnecký et al. [19] describe quality control and assurance of IC analysis. After the experiment, samples of soil and SC were air dried to constant weight. A part was then analyzed for PTE contents by total digestion. For comparison, a blank experiment was performed for each leaching (as described but without SC sorbent).

The following equation was applied to determine:

Sorption ratio between PTE on SC and PTE in solution after leaching =

$$\frac{Aa-Ab}{sol1-sol0}$$ (1)

Where **Aa** is PTE content in SC after leaching, **Ab** is PTE content in SC before leaching, **sol**^**1**^ is PTE content in solution after leaching, **sol**^**0**^ is PTE content in a blank solution.

#### Desorption

After drying, a part of SC (1g) was analyzed for total PTE content, and 3 g was prepared in a second column experiment. The arrangement was similar to the first column leaching experiments except that the soil sample was absent. The purpose of the second leaching was to test the performance of SC at retaining PTE after leaching with an acidified DI_H2O_ solution (adjusted to pH 3.4 using HNO_3_). Experimental solvent was introduced by a peristaltic pump set to flow at 100 mL/hr for 5 continuous hours. Leachate was collected every 30 minutes, and then later analyzed for PTE content.

## Results and Discussion

### Properties of solid samples

Soil samples O and AB were both acidic, with little difference in pH between these two layers (Table 1). The amount of combustible organic matter was considerably higher in soil O (77.0%) than in soil AB (22.4%). NCS analysis estimates the percentage of C in soil O as 28.1%, and 4.6% in soil AB. Content of N was also higher in soil O, a probable reflection of the amine groups associated with humic and fulvic acids. Both soils contained relatively poorly polymerized organic substances. CEC of soil O was considerably higher than that of soil AB.

Acid digestion of soil showed that heavy metals Cd and Pb were most enriched in soil O, whereas As was more concentrated in soil AB (Table 1). The observed soil PTE concentrations were well in excess of typical guideline values for these elements. The content of PTE in SC was measured in order to obtain values for Ab Eq 1; traces of As, Cd and Pb were observed on the shredded card. These values are likely to vary according to the source of cardboard, but are negligible compared to the concentrations in contaminated soil.

### Leaching experiments (adsorption)

#### pH dynamics

**Fig 1:**
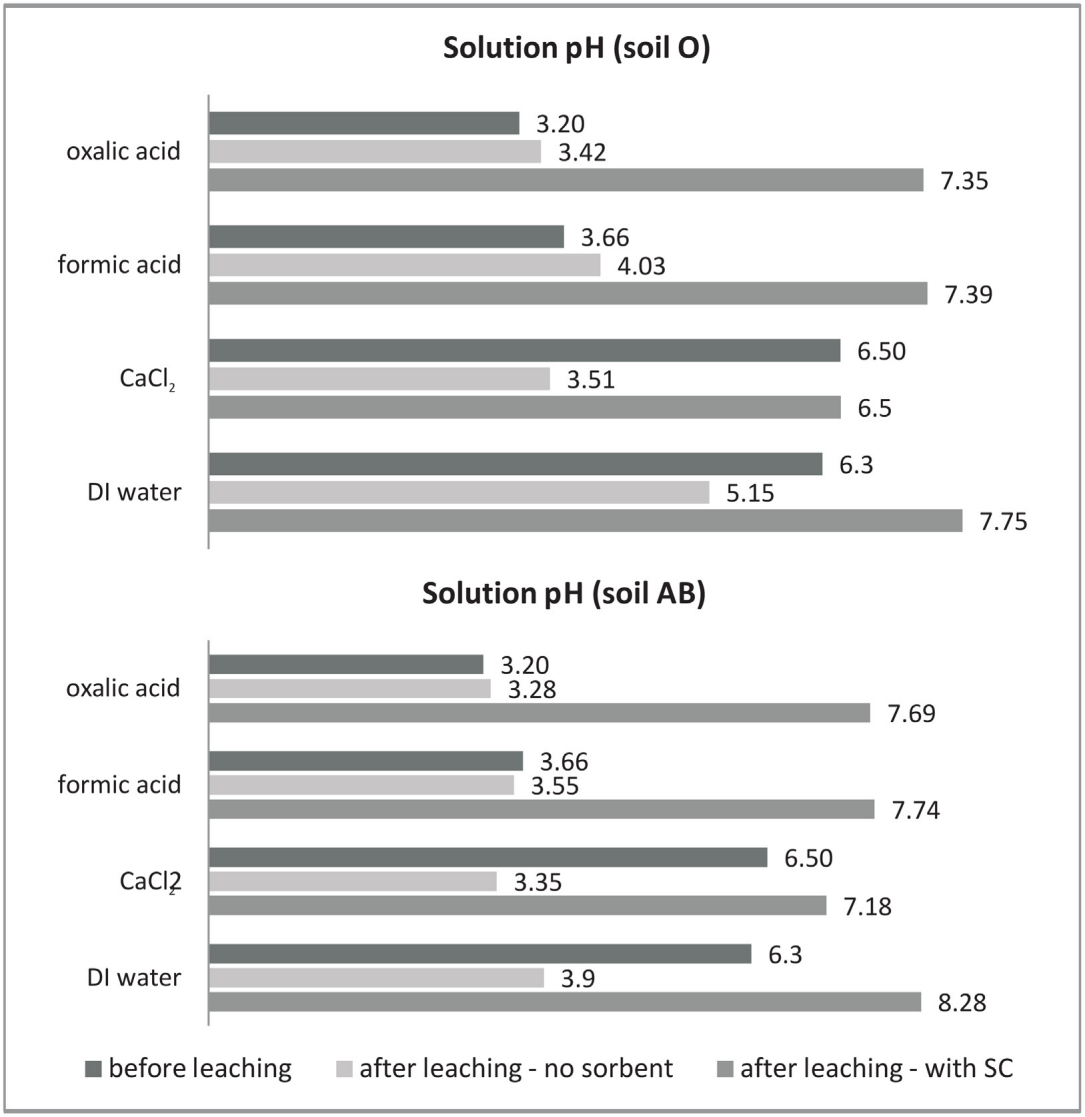
Change in solution pH before and after first leaching when shredded card sorbent was either present or absent in the leaching column.

The alkaline nature of SC (pH >8.0), as well as its light open structure, and ion exchange surfaces are reasons for its application in this study as a potential sorbent of metal ions. pH measurements before and after leaching (Fig 1) showed that significant changes occurred to solution pH when SC was present in the column. Largest pH shifts occurred in oxalic acid (+4.49 and +4.15 pH units for AB and O samples respectively). Highest solution pH values after leaching were observed for AB samples. This is a probable reflection of the initially lower pH of soil O, and a higher content of reactive humus. There was a greater water extractable content of organic acids from soil O than for soil AB (Table 2), which can buffer solution against further pH increases.

**Table 2:** Content of main anions in leachate solution (mg/L) after leaching.

Leaching experiment	formate	oxalate	lactate	acetate	NO_3_^-^	SO_4_^2-^	PO_4_^3-^
**Soil O**	mg/L						
oxalic—no sorbent	0.099	87.08	0.246	0.154	0.179	1.235	2.855
oxalic—with SC	0.080	1.499	0.613	0.418	0.241	1.833	b.d.l
formic—no sorbent	43.22	0.708	0.160	0.185	0.324	0.549	0.663
formic—with SC	41.64	1.015	0.570	0.660	0.065	1.170	b.d.l
CaCl_2_—no sorbent	b.d.l	0.146	b.d.l	b.d.l	0.920	0.619	0.284
CaCl_2_—with SC	b.d.l	0.375	b.d.l	b.d.l	0.420	1.105	b.d.l
DI_H2O_ - no sorbent	b.d.l	0.238	0.109	0.034	0.290	0.677	0.613
DI_H2O_ - with SC	0.045	0.931	0.140	0.057	0.052	1.151	0.157
**Soil AB**							
oxalic—no sorbent	0.109	84.74	0.126	0.085	2.962	0.942	2.625
oxalic—with SC	b.d.l	0.526	0.245	0.050	4.895	1.713	b.d.l
formic—no sorbent	43.65	0.415	0.206	0.126	3.719	0.381	0.619
formic—with SC	41.53	0.242	b.d.l	b.d.l	5.610	0.939	b.d.l
CaCl_2_—no sorbent	b.d.l	0.064	b.d.l	b.d.l	10.63	0.517	0.183
CaCl_2_—with SC	b.d.l	0.255	b.d.l	b.d.l	5.014	1.092	b.d.l
DI_H2O_ - no sorbent	b.d.l	0.075	b.d.l	b.d.l	15.86	0.278	0.411
DI_H2O_ - with SC	b.d.l	0.028	b.d.l	b.d.l	6.659	1.129	b.d.l

b.d.l = below determination limit

#### Anion content in leachates

In some cases, there was an increase in LMMOA (oxalate, lactate, and acetate) and NO_3_^-^ and SO_4_^-^ concentration in leachate for samples with SC present (Table 2). A simple extraction was made on SC to determine the content of water extractable anions (1 g SC: 100 mL DI_H2O_, with 4 hrs shaking). Results showed that SC is a source of trace amounts of the following anions: oxalate (130 mg/kg), lactate (540 mg/kg), acetate (592 mg/kg), NO_3_^-^ (140 mg/kg), SO_4_^2-^ (427 mg/kg). Consequently, under certain leaching conditions, small increases in the aforementioned anions could be expected along with the studied PTE.

#### Comparison of PTE content in solution after leaching with and without SC

**Fig 2:**
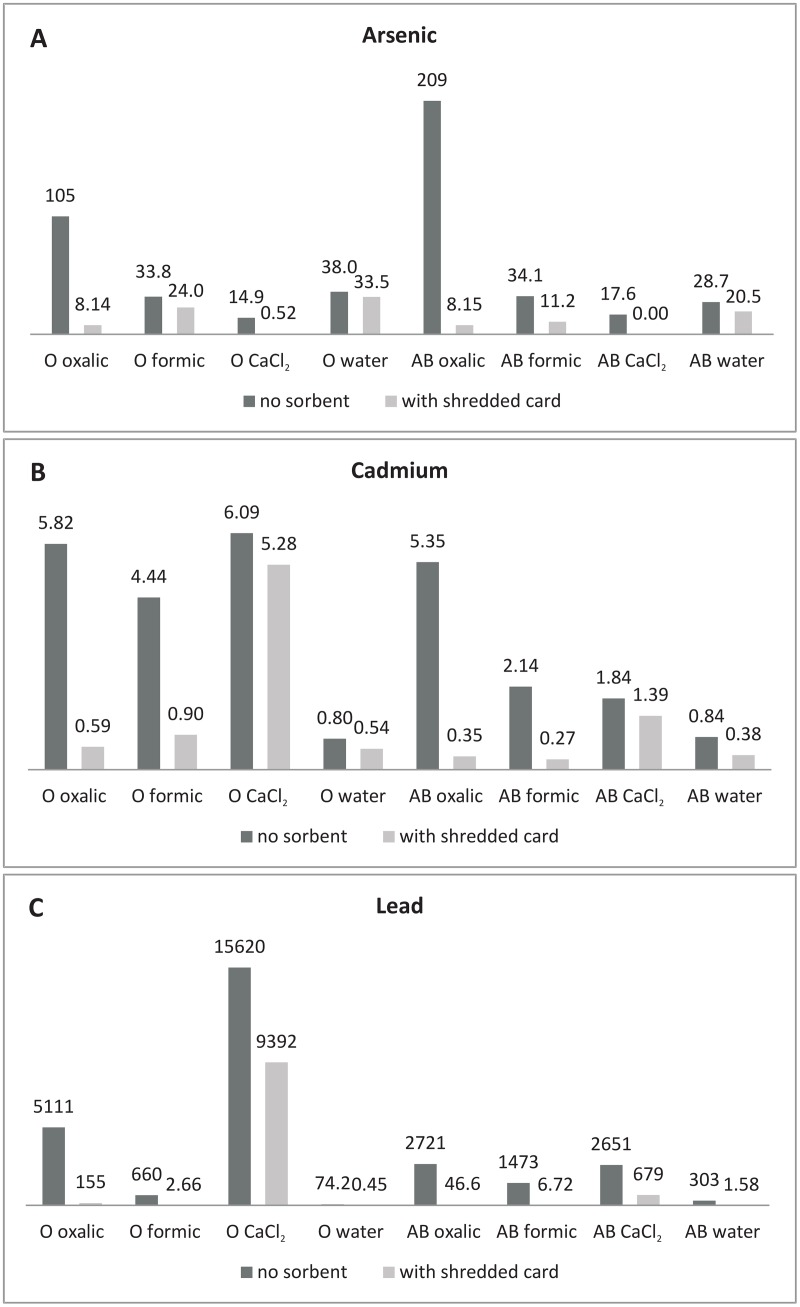
Difference in solution concentrations of As (A), Cd (B), Pb (C) after 24hrs of leaching (mg/kg) when shredded card sorbent was either present or absent in the leaching column.

Fig 2 illustrates the data for measured PTE concentrations in solution after leaching with and without SC present in the column. Ratios of PTE content in solution without SC to those with SC are also shown in Table 3.

**Table 3:** PTE content in solution without shredded card filter as a ratio of the PTE content in solution with shredded card filter.

	As	Cd	Pb
**soil O**	PTE content ratio		
oxalic with SC < oxalic—no sorbent	12.9	9.86	33.0
formic with SC < formic—no sorbent	1.41	4.93	248.1
CaCl_2_ with SC < CaCl_2_—no sorbent	28.7	1.15	1.66
DI_H2O_ with SC < DI_H2O_ - no sorbent	1.13	1.48	164.9
**soil AB**			
oxalic with SC < oxalic—no sorbent	25.6	15.3	58.4
formic with SC < formic—no sorbent	3.05	7.93	219.2
CaCl_2_ with SC < CaCl_2_—no sorbent	*b.d.l	1.32	3.90
DI_H2O_ with SC < DI_H2O_ - no sorbent	1.39	2.21	191.8

*b.d.l = below determination limit for the sample with added SC sorbent

Arsenic: Comparison of As content in solution for columns with/without SC indicates a high level of As sorption by SC from some of the studied solutions (Fig 2). The sorbent is particularly effective at immobilizing As from solutions of oxalic acid and CaCl_2_. Far less As sorption was observed when formic acid and DI_H2O_ were applied. Table 3 confirms the difference between As content in solution after leaching with/without SC between oxalic acid and formic acid. Formic acid is a simple mono-carboxylic acid of approximately half the molecular mass of oxalic acid (di-carboxylic), which may contribute to lesser interception of As-formate complexes. Regarding differences between As sorption in O or AB samples, it is evident from columns without SC that, with the exception of DI_H2O_, experimental solvents released a greater amount of As from soil AB than from soil O. Moreover, the sorption efficiency was greater for As released from soil AB than for soil O. Studies have shown that As is highly reactive with natural organic matter, and that soil organic matter and dissolved organic carbon (DOC) content correlate strongly with As sorption [20]. This has led to conflicting theories that organic matter can either immobilize As due to strong retention at the stationary solid phase, or can in fact enhance As transport via soluble DOC-As complexes. Our results suggest the latter. A greater source of fulvic, humic, and simple organic acids is likely to aid As transport from soil O. The higher portion of As interception by SC for AB samples may be partly pH dependent. The difference in solution pH before and after leaching was higher in every case for AB samples; pH of DI_H2O_ reached 8.28 after leaching with SC present. Arsenic forms a variety of inorganic and organic compounds in soils, but most of them are in the inorganic forms of As(III) and As(V) [21], and although complex anions (AsO_2_^-^, AsO_4_^3-^, HAsO_4_^2-^, and H_2_AsO_3_^-^) are the most common mobile forms of As, sorption occurs in the pH range from 7–9 [22].

The order of As sorption (Table 4) was the same for both soil samples (CaCl_2_ > oxalic > formic > DI_H2O_). As previously noted, sorption of As by SC is much greater in oxalic acid than for formic acid. Arsenic was most strongly sorbed from CaCl_2_, with a SC to solution ratio of >14:1 in the case of soil AB. This also reflects the direct observations, whereby experimental solution without SC contained up to 28x more As. In contrast, for metals Cd and Pb, CaCl_2_ was the most effective solvent with very little of these elements bound by the SC. The reason may be attributable to the release mechanism of heavy metals by CaCl_2_, which is the exchange between Ca^2+^ and divalent cations, whereas As exists primarily as complex anions. Arsenic may react with CaCl_2_ to form calcium arsenate, a soluble polar compound that could bind to SC via the positive portion. In DI_H2O_, a considerably smaller amount of As was sorbed onto SC; very low ratios were obtained for both soil samples. Weak sorption of As from DI_H2O_ could be due to the low ionic strength of DI_H2O_.

**Table 4:** As, Cd, Pb concentrations in SC after leaching (top), and ratio between PTE on SC and PTE in solution after leaching (bottom).

	PTE content in SC after leaching					
	**Aa–Ab** (mg/kg)					
	**As**		**Cd**		**Pb**	
	**O**	**AB**	**O**	**AB**	**O**	**AB**
Oxalic acid	58.1	43.7	3.18	1.32	2459	647
Formic acid	7.35	3.69	2.98	1.11	456	700
CaCl_2_	6.17	14.4	0.32	0.16	1415	484
DI_H2O_	0.80	1.07	0.25	0.31	25.2	14.3
	Ratio between PTE on SC and PTE in solution after leaching					
	(higher ratio = greater sorption)					
	**As**		**Cd**		**Pb**	
	**O**	**AB**	**O**	**AB**	**O**	**AB**
Oxalic acid	7.09	5.31	5.15	3.37	15.8	13.7
Formic acid	0.29	0.29	3.16	3.59	169	103
CaCl_2_	11.1	14.0	0.03	0.01	0.15	0.70
DI_H2O_	0.01	0.03	0.20	0.45	40.3	4.58

**Aa** is PTE content in SC after leaching (mg/kg), **Ab** is PTE content in SC before leaching (mg/kg) Eq 1

Cadmium: A considerable amount of Cd was retained on SC after leaching for experiments involving oxalic and formic acids; ≤ 3.18 and 2.98 mg/kg Cd respectively (Table 4). This is not just a reflection of the amount of Cd that was mobilized by the organic acids, because CaCl_2_ leached a comparable amount of Cd (Fig 2). SC sorbent can be considered particularly effective at immobilizing Cd from the studied LMMOA as shown by the ratio between Cd concentration in solutions with and without SC present in the column (Table 3). The greatest difference was observed for oxalic acid with soil AB, whereby 15.9x more Cd was detected in leachate from the column without SC. Similar concentrations of Cd were released into oxalic acid from soils O and AB (5.82 and 5.35 mg/kg respectively), but greater interception of Cd by SC occurred for soil AB. Of the studied PTE, sorption from CaCl_2_ was the lowest for Cd. Lodenius [23] studied the leaching of Cd using salt solution (pH 7.0); strong leaching was achieved by neutral solution containing Ca^2+^. It is also stated by Kabata-Pendias [22], that in some cases, Cd adsorption can decrease in the alkaline range due to the competition from Ca^2+^ ions.

Cd Sorption ratios (Table 4) reflect the direct observations for the most part. In soil O, the order of SC sorption was repeated; oxalic > formic > DI_H2O_ > CaCl_2_. In AB sample there is some ambiguity. The largest difference between leachate Cd concentration for experiments with/without SC was observed for oxalic acid. However, the SC sorption ratio suggests that sorption of Cd was slightly higher in formic acid. Nevertheless, we can state that SC was effective at immobilizing Cd in both oxalic and formic acid solutions. Similar to the direct observations, sorption of Cd onto SC was very weak when CaCl_2_ was used (SC:leachate ratios of 0.03:1 and 0.01:1 for O and AB samples respectively). A small proportion of Cd was bound to SC in the DI_H2O_ experiment (ratio < 1:1). In addition to the low ionic strength of DI_H2O_, poor sorption could result from generally low concentrations of Cd in solution.

Lead: Total Pb soil content averaged 24,962 and 5,046 mg/kg in O and AB samples respectively. The extremely high loading with Pb means that its distribution is likely to be heterogeneous. This was evident even at the sample level. Consequently, it is difficult to draw conclusions based on differences in soil concentrations before and after leaching. However, despite the deviations in total Pb soil concentrations, there is a clear difference in solution Pb content after leaching when SC sorbent was present (Fig 2). Compared to As and Cd, the difference in Pb solution concentration with and without SC was much higher, except for in CaCl_2_ (Table 3). Although the amount of Pb released from O and AB samples into formic acid was far lower than that of oxalic acid, the proportional difference of Pb in solution between samples with or without SC was much greater (SC was more effective at immobilizing Pb in formic acid). Lead reacts with oxalic acid to produce lead oxalate, which is insoluble, however, with excess oxalate ions the soluble complex ion Pb(C_2_O_4_)_2_^2-^ is formed. The anionic nature of Pb(C_2_O_4_)_2_^2-^ means that it is unable to form electrostatic bonds with the negatively charged surface of SC, thus as experiment time progresses there is less interception of Pb-oxalate by SC. This may go some way to explaining why the sorption of Pb was less for oxalic acid. The result may also be a reflection of the dissociation of these acids (oxalic acid = 1.25_pKa1_, 3.81_pKa2_, formic acid = 3.75_pKa_). Moreover, formic acid is mono-carboxylic and two formate ions are needed to form a neutral complex with Pb^2+^, which may explain the increased sorption onto negatively charged SC. Perelomov et al. [24] suggest that such binding is attributable to the formation of heavy metal-organic acid anion complexes and the simultaneous sorption of acids at positively charged sites with the formation of mineral–organic compounds. Table 2 shows that in samples with SC, almost no oxalate is present in leachates, whereas in the case of formate the amount is almost equal to that of experiments without SC. This prompts the suggestion that formate is acting as a Pb carrier and donor to the SC as it passes, rather than forming a stable organo-Pb complex. We also observed a substantial decrease of Pb in the DI_H2O_ leachate of column experiments with SC present; 164 and 191x more Pb was in DI_H2O_ leachate without SC for O and AB samples respectively. Results were comparable to those of Battaglia et al. [25] who studied Cd and Pb sorption in a pH independent batch experiment using paper mill sludge admixtures with soil sample. After 15 days of wetting, the amount of Pb retained by soil-paper mill sludge had doubled compared to a control. Cadmium on the other hand was not overly influenced by the addition of paper mill sludge. Of all the studied solvents, the highest pH after leaching with and without SC was observed for DI_H2O_. He et al. [26] also observed considerable increases in Pb^2+^ sorption in a paper sludge amended soil, which correlated with the rise of pH during the sorption process. This has quite significant implications. Studies have shown increasing fiber surface charge with increasing pH, consistent with the expected dissociation of carboxylic groups [10]; hence, an increased sorption of cations.

Lead sorption ratios (Table 4) confirm the high interception rate of Pb in formic acid, a high removal of Pb also in DI_H2O_, followed by oxalic acid, and poor sorption of Pb from CaCl_2_. There are some discrepancies concerning the order of Pb sorption in the studied solutions for AB sample. The SC sorption ratio suggests that in AB sample, greater Pb sorption efficiency occurred in oxalic acid than in DI_H2O_.

### Leaching experiments (desorption)

**Fig 3:**
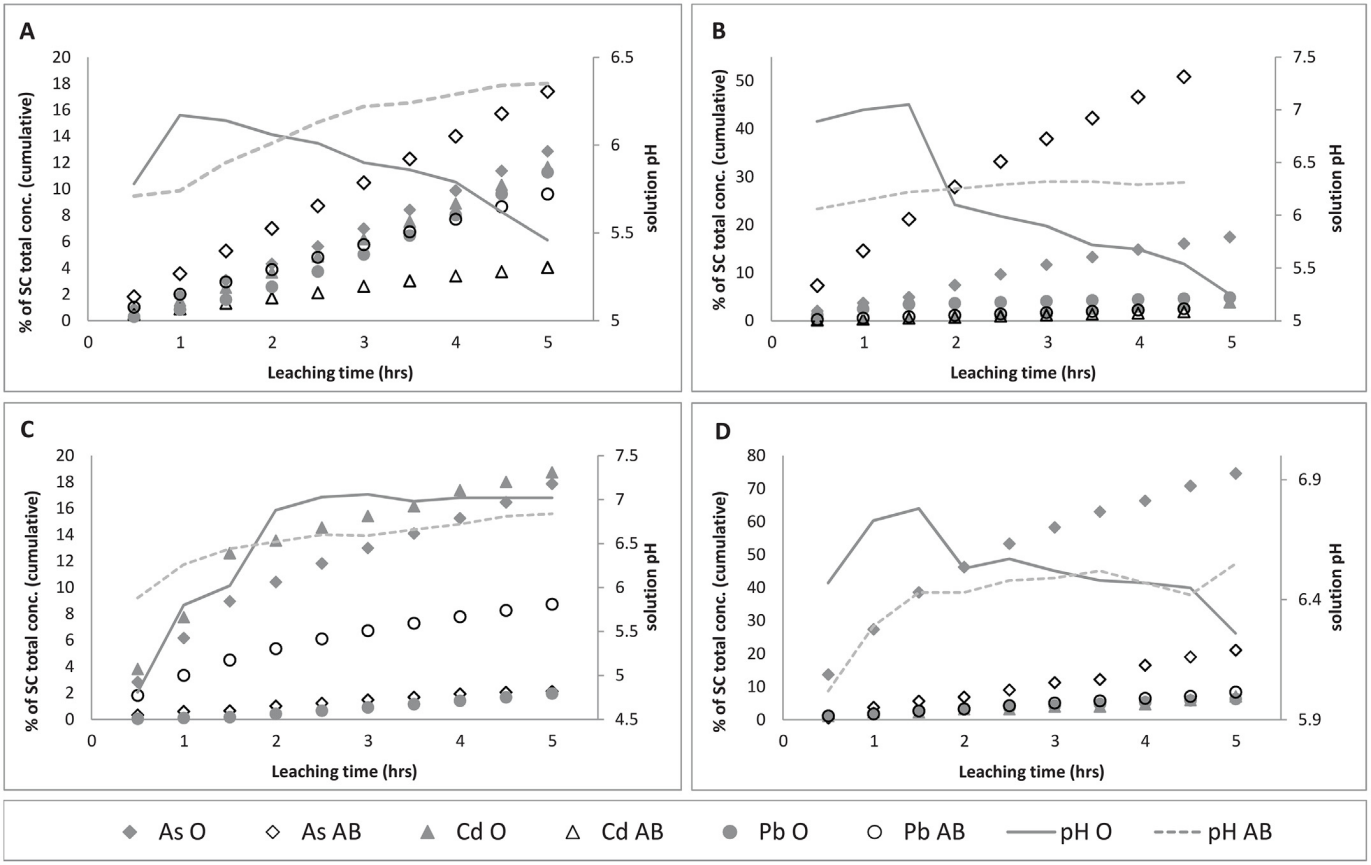
Desorption of As, Cd, Pb from SC after leaching with acidified (pH 3.4) deionized water. Desorption trends from SC, where initial sorption of studied PTE onto SC was in solutions of oxalic acid (A), formic acid (B), CaCl_2_ (C), deionized water (D).

The ability of SC to retain the studied PTE varied widely, as indicated in Fig 3. Solution (leachate) concentrations were converted from mg/L to mg/kg based on hourly extraction ratios. PTE solution concentrations (mg/kg) at collection intervals were then taken as a proportion of total PTE content on SC prior to desorption.

Arsenic: Arsenic was the PTE most readily released from SC for all sorption pathways except for CaCl_2_, whereby Cd desorption was slightly higher. Significant differences in the extent of release were observed according to the type of initial sorption and the soil layer from which As originated. Significant differences between amounts of PTE released from SC, when initial source of the PTE was from either O or AB soil samples, were validated by t-test (Table 5). Arsenic desorption from SC was between 10–20% for samples with As initially released from soil sample O, and sorbed from oxalic and formic acid (Fig 3A and 3B). However, for AB samples, desorption was > 50% for samples initially sorbed from formic acid. This confirms the stronger binding of As to SC from oxalic acid compared to formic acid, as identified during the adsorption experiment (Tables 3 and 4). Because the sorption efficiency was high for CaCl_2_, it is therefore also expected that desorption would be relatively low. This is certainly the case for AB sample (~2%), but for soil O, a greater release of As occurred (~18%). The highest release of all PTE was observed for As with initial sorption from DI_H2O_ (>70% desorption). This also reflects the low level of initial sorption observed in adsorption experiments. It leads to the inference that SC may not be effective at retaining As if left exposed to the elements and not immediately disposed of.

**Table 5:** Significant differences (independent t-test) between PTE released from SC after sorption from sample O or sample AB in different leachates.

	As		Cd		Pb	
	t	p	t	p	t	p
Oxalic acid	6.979	<0.001*	9.296	<0.001*	1.123	0.276
Formic acid	8.549	<0.001*	4.635	<0.001*	1.377	0.185
CaCl_2_	5.822	<0.001*	-	-	4.383	<0.001*
DI_H2O_	4.068	<0.001*	-	-	2.110	0.049

* Significant at 95% confidence level

Cadmium: For SC samples with initial sorption by oxalic acid, Cd desorption was low (soil AB) to moderate (soil O), with ~4% and ~11% Cd released respectively. Furthermore, Cd release for soil O was linear, whereas for AB samples Cd release decreased after approximately 3 hrs. For SC samples with initial Cd sorption in formic acid, greater Cd desorption was again from O samples, but only marginally. After 5 hrs of leaching, Cd release for both O and AB samples was relatively small, 3.8 and 2.0% respectively. Again, we suggest that because sorption onto SC was generally higher for Cd from soil AB, the subsequent rate of release was lower (Fig 3A and 3B). Solution pH seems to have little influence on the release of Cd in samples with initial sorption from formic acid. The released Cd remained low for both samples. For SC with initial sorption from CaCl_2,_ Cd was the element most readily released, and again reflects the very low initial sorption. For soil AB, the concentrations were below detection limit. For SC with initial sorption from DI_H2O_, desorption of Cd remained low for the duration of the experiment (<5% released), and for soil AB, Cd was below detection level.

Lead: Pb featured among the least leached elements in the majority of desorption experiments, which is promising for the potential application of SC as a post soil washing filter. For SC samples with initial Pb sorption from oxalic acid, a modest release of Pb occurred, up to ~6% and 11% after 5 hrs of leaching for soils AB and O respectively. Release of Pb from samples with initial sorption in formic acid was low overall for both O and AB samples (~4.8% and 2.5% for O and AB respectively). The retention of Pb for these samples is highly significant, as shown by the adsorption efficiency (Table 3). During the adsorption experiment, SC intercepted a high proportion of Pb, despite the extracting strength of CaCl_2_ toward divalent cations. In the desorption experiment, approximately 2% and 8.7% of Pb was released from SC for soil O and soil AB respectively. This limited leaching of Pb may be a reflection of the buffering against pH decrease in this sample (Fig 3C). With regard to the leaching of Pb from SC that was initially adsorbed in DI_H2O_, the results indicate good retention (Fig 3D). After 5 hrs of leaching, approximately 8.5 and 6.5% of total Pb was released from SC for AB and O samples respectively.

## Conclusions

Increases in solution pH (as much as pH +4.49) were observed after leaching through SC. Sorption of PTE onto SC varied between the studied PTE, and according to type of leaching solution. In oxalic acid, all of the studied PTE were effectively sorbed. In formic acid, Pb sorption was very efficient, but As and Cd were adsorbed to a lesser extent. In DI_H2O_, only Pb showed a high rate of sorption. A generally greater degree of sorption was achieved for PTE that had leached from a mineral soil layer. Further attention is needed to clarify the role of DOC, and the influence that degree of humification of source soil has on PTE sorption. Desorption experiments showed that release of PTE from SC varies according to the source of the PTE (organic vs. mineral soil), and the type of solvent. The soil layer from which PTE are washed not only dictates the amount of sorption (respective of total contents), but also has an effect on the subsequent release of the PTE; a greater adsorbed proportion of PTE generally results in lower release of that element. Arsenic was the PTE most readily leached in all desorption experiments. A high rate of Cd sorption from LMMOA solutions was followed by strong retention during desorption experiments. For sorption of Cd onto SC from DI_H2O_, the desorption experiment indicated a good level of retention. For SC samples with initial Pb sorption from LMMOA and CaCl_2_, desorption in acidified water was relatively low (≤ 10% after 5 hrs of leaching). SC was able to retain Pb after adsorption in DI_H2O_, with losses of no more than 8.5% of total lead. Shredded card showed good potential as a sorbent of toxic elements in various solutions.

## Supporting Information

S1 Fig Map showing where soil was collected, in the forested area adjacent to a lead processing plant. (JPG)

S2 Fig Image of shredded card as it was used in leaching experiments (top), and the experiment arrangement (bottom). (JPG)
