# Supplementary figures and images for: Sustainable Soil Washing: Shredded Card Filtration of Potentially Toxic Elements after Leaching from Soil Using Organic Acid Solutions
Source: PLoS One. 2016 Feb 22;11(2):e0149882. doi: 10.1371/journal.pone.0149882 (PMC4765769; doi:10.1371/journal.pone.0149882)

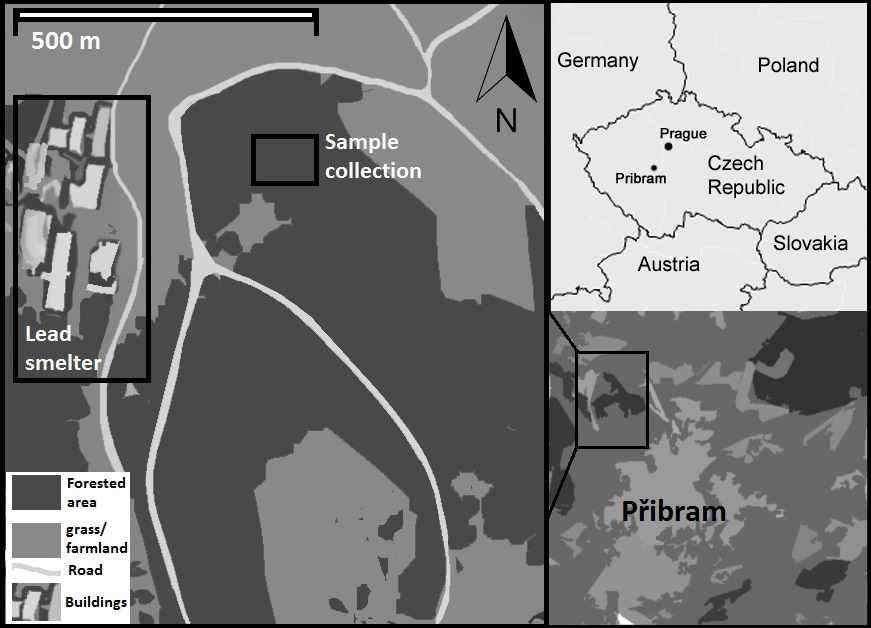

Supplement: S1 Fig — (JPG) [file pone.0149882.s001.jpg]

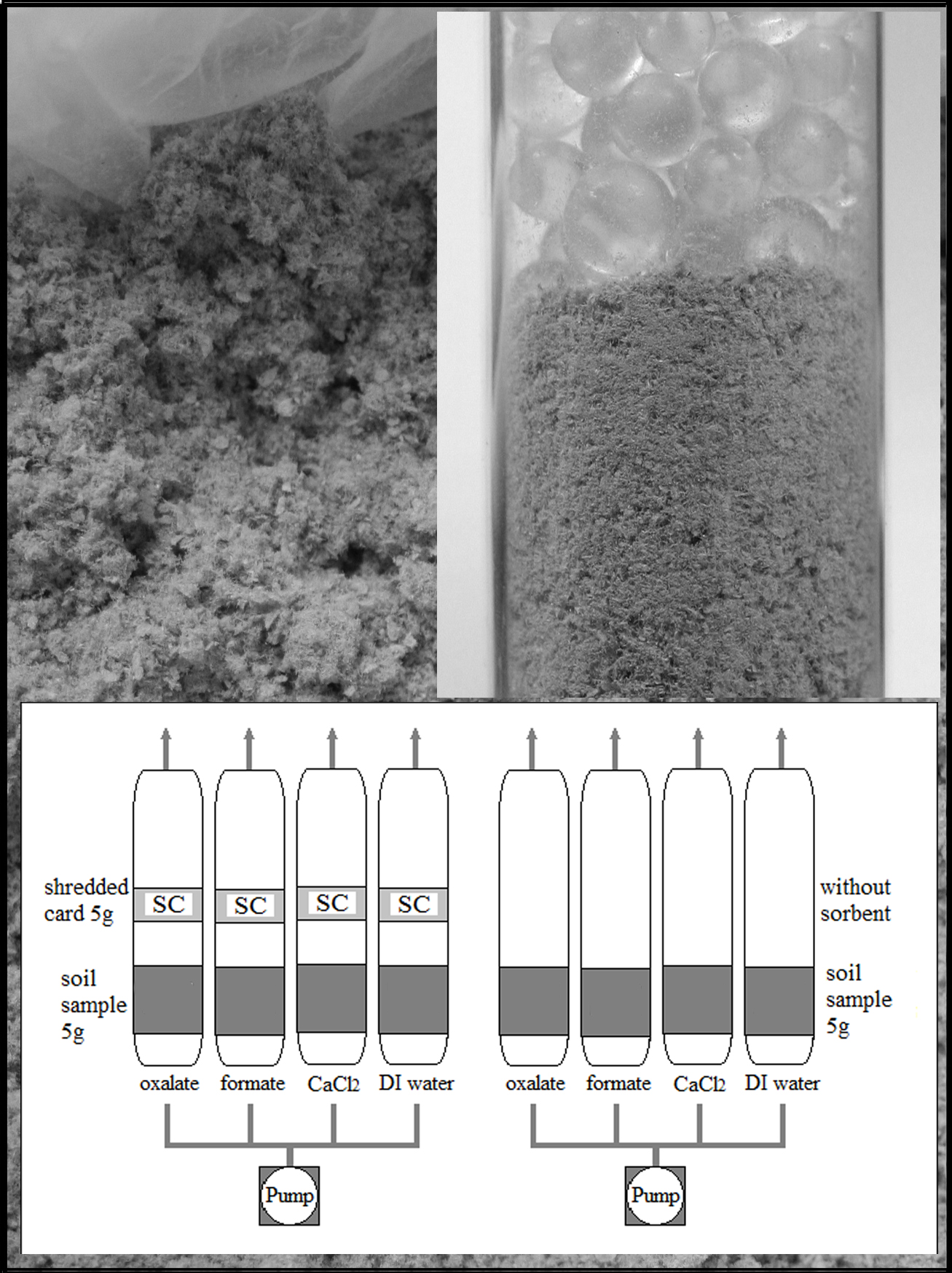

Supplement: S2 Fig — (JPG) [file pone.0149882.s002.jpg]
